# The Multifaceted Functions of Plant Asparagine Synthetase: Regulatory Mechanisms and Functional Diversity in Growth and Defense

**DOI:** 10.3390/plants15030362

**Published:** 2026-01-24

**Authors:** Gang Qiao, Siyi Xiao, Jie Dong, Qiang Yang, Haiyan Che, Xianchao Sun

**Affiliations:** 1 College of Plant Protection, Southwest University, Chongqing 400715, China; q51306013020@email.swu.edu.cn (G.Q.); xiaosiyi@email.swu.edu.cn (S.X.); yq2192840164@email.swu.edu.cn (Q.Y.); 2 Key Laboratory of Pests Comprehensive Governance for Tropical Crops, Ministry of Agriculture and Rural Affairs, Haikou 571101, China; 3 Key Laboratory of Agricultural Biosafety and Green Production of Upper Yangtze River (Ministry of Education), Southwest University, Chongqing 400715, China

**Keywords:** asparagine synthetase, nitrogen transport, abiotic stress, stress and disease resistance, genetic breeding

## Abstract

Asparagine synthetase (AS) is a key enzyme in plant nitrogen metabolic network. Beyond its canonical role as a major nitrogen transport and storage molecule, asparagine also serves critical functions in plant immunity and tolerance to environmental stresses. This review systematically summarizes the characteristics of the core AS-mediated asparagine biosynthesis pathway and two other minor pathways in plants. It details the distribution of the *AS* gene family, protein structure, and evolutionary classification. The mechanisms governing AS expression are analyzed, revealing tissue-specific patterns and precise regulation by nitrogen availability, abiotic stresses, and exogenous hormones, mediated through an interactive network of cis-acting elements and transcription factors. Furthermore, the biological functions of AS are multifaceted: it influences plant biomass and nitrogen use efficiency by regulating nitrogen uptake, transport, and recycling during growth and development; it contributes to abiotic stress tolerance by synthesizing asparagine to maintain cellular osmotic balance and scavenge reactive oxygen species; and it indirectly enhances antibacterial and antiviral capacity by activating the SA signaling pathway and modulating programmed cell death. Current knowledge gaps remain regarding the crosstalk between AS-mediated signaling pathways, the upstream transcriptional regulatory network, and the balance between nitrogen utilization and disease resistance in crop breeding. Future research aimed at addressing these questions will provide a theoretical foundation and molecular targets for improving crop nitrogen use efficiency and breeding resistant cultivars.

## 1. Introduction

Nitrogen is essential for plant growth, influencing plant development and crop yield. Within the complex nitrogen metabolic network, the efficient assimilation, long-distance transport, and effective remobilization of nitrogen are critical physiological processes. Among various nitrogen carriers, asparagine (Asn) stands out due to its high nitrogen-to-carbon ratio and remarkable stability, making it a principal molecule for nitrogen transport via the phloem and nitrogen storage in many plant species [1,2].

The biosynthesis of Asn in plants is primarily catalyzed by AS, which utilizes energy derived from ATP hydrolysis to transfer the amide group from glutamine (Gln) to aspartate (Asp), yielding Asn and Glu (Glu) [3]. AS is a catalytic enzyme that is ubiquitously present in plants, animals, and microorganisms. Among its isoforms, AS-B is uniquely found in plants [4,5]. This enzyme utilizes the energy from ATP hydrolysis and employs glutamine as nitrogen sources to convert aspartate and glutamine into Asn and Glu [6,7,8]. Recent research on As in plants has predominantly centered on three key areas: (i) nitrogen mobilization during seed germination [9,10]; (ii) nitrogen transport and redistribution during plant growth [11]; (iii) nitrogen remobilization between sink and source tissues during plant senescence [1,12]. These findings underscore the pivotal role of AS in the transport and distribution of nitrogen throughout plant development. Although current research indicates that AS possesses these diverse functions, many underlying mechanisms remain unclear. The specific molecular mechanisms of AS in plant responses to abiotic stress are not fully elucidated, and emerging reports on its role in antiviral defense similarly lack detailed molecular explanations.

**Figure 1:**
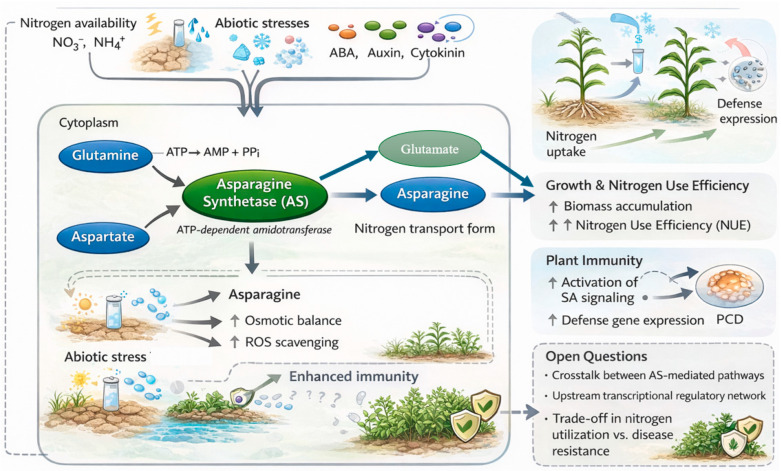
Schematic diagram of the As-mediated regulatory network in nitrogen metabolism, stress tolerance, and immunity in plants. This figure was generated using AI tools (ChatGPT-4o) and manually refined to ensure scientific accuracy. Nitrogen Signaling: The NLP transcription factors (e.g., NLP7) directly activate ASN genes in response to nitrate/ammonium status. ABA Stress Signaling and ABRE/AREB Elements: Induction of ASN expression (e.g., TaASN1, MaAS) under drought and osmotic stress via ABA-dependent pathways and promoter ABRE elements is documented. ROS Signaling: H_2_O_2−_induced ASN expression (e.g., NbASN) and the role of AS in redox homeostasis through glutathione synthesis are established. SA Signaling: The catalytic product glutamate (Glu) from AS serves as a direct substrate for the ICS pathway of SA biosynthesis, linking AS activity to systemic acquired resistance. Growth vs. Stress Switch: The dual role of AS in promoting growth under optimal conditions and redirecting nitrogen to stress tolerance under adversity is supported by phenotypic analysis of mutants and overexpression lines across species.

This review aims to synthesize current knowledge on the multifaceted functions of plant As, with a specific focus on the AS-B isoform. We will begin by outlining the enzymatic pathways of asparagine synthesis and the structural classification of AS enzymes. We will then delve into the complex transcriptional regulation of *AS-B* genes. A major focus will be placed on discussing the emerging roles of AS-B in coordinating plant development, abiotic stress tolerance, and biotic stress resistance. Finally, we will highlight critical unresolved questions and propose future research directions to unravel the sophisticated mechanisms by which this key metabolic enzyme orchestrates the plant growth-defense balance. Synthesizing this information is crucial for exploring the potential of AS-B as a strategic target for breeding crops with enhanced nitrogen use efficiency and resilience (Figure 1).

## 2. Biosynthesis Pathways of Asparagine in Plants

The biosynthesis of Asn in plants primarily occurs through three pathways: (1) The primary route involves AS, which is ATP-dependent and transfers the amide group from glutamine or ammonium to aspartate, generating Asn and Glu. This pathway is the major source of Asn in plants [13]. In this process, AS-B first activates the amide group of Gln, which is then transferred to the amide group of Asn via AS, ultimately forming Glu and Asn. The Asn produced via this pathway has a high nitrogen-to-carbon ratio and is chemically stable, serving as the core form for long-distance nitrogen transport within the plant and is translocated via the phloem to young tissues to support growth requirements. (2) The cyanide detoxification-associated pathway: Cyanide reacts with cysteine under the catalysis of β-cyanoalanine synthase to produce β-cyanoalanine, which is then hydrolyzed by β-cyanoalanine hydratase to yield Asn and a small amount of ammonia. The primary function of this pathway is to detoxify accumulated cyanide in plants, rather than being a major synthesis route for Asn [14]. (3) The reversible transamination reaction in peroxisomes: Asn and a 2-oxoacid react under the catalysis of asparagine-oxoacid transaminase to produce 2-oxosuccinamate and another amino acid. This process occurs in peroxisomes and is hypothesized to be related to photorespiratory nitrogen metabolism; it is a reversible reaction. However, minimal Asn in plants is synthesized via this route. Plant Asn synthesis predominantly relies on the first pathway, with the latter two playing minor roles only under specific physiological conditions [2].

## 3. Structure and Classification of Asparagine Synthetase

AS is distributed in both prokaryotes and eukaryotes [15,16], typically encoded by small gene families and widely present in plants [17,18]. AS genes have been successively reported in various plants: three in Arabidopsis thaliana, two in rice (*Oryza sativa*), five in wheat (*Triticum aestivum*), two in tomato (*Solanum lycopersicum*), and four in *Nicotiana benthamiana*, with corresponding genes identified in other species (C. Liu [19]. Based on structural characteristics, AS is mainly classified into two types: ammonium-dependent AS-A (EC 6.3.1.1) and glutamine-dependent AS-B (EC 6.3.5.4) (Lomelino et al., 2017 [3]; Shi et al., 1997 [20]). AS-A is primarily found in prokaryotes, while AS-B is distributed in both prokaryotes and eukaryotes. AS-A is encoded by the *asnA* gene, uses NH_4_^+^ as the sole amide donor and ATP as the energy source, and synthesizes Asn from aspartate. In contrast, AS-B, encoded by the *asnB* gene, can utilize either NH_4_^+^ or Gln to synthesize Asn.

The AS-B protein consists of two domains that coordinate to complete the catalytic reaction: an N-terminal glutamine amidetransferase domain and a C-terminal synthetase domain. The glutamine amidetransferase domain is primarily responsible for recognizing and binding glutamine, dissociating its amide group, and transferring it to the catalytic center. This functionality is a key feature distinguishing AS-A from AS-B. The synthetase domain primarily provides binding sites for ATP and aspartate, supplies energy for the amide group transfer, receives the amide group, and ultimately facilitates the formation of reaction products. Currently cloned AS-B genes from plants show high amino acid sequence homology, reaching approximately 80% or even higher (Lomelino et al., 2017) [3], with the highest homology observed in the glutamine amidetransferase and synthetase domains. All plant AS-B proteins contain the glutamine amidetransferase domain, hence they are classified as glutamine-dependent AS-B (Lam et al., 1994) [21]. The synthetase domain contains three conserved residues, Cys1, His101, and Asp29, which participate in the transamination function involving Gln (Mei & Zalkin, 1989 [22]).

**Figure 2:**
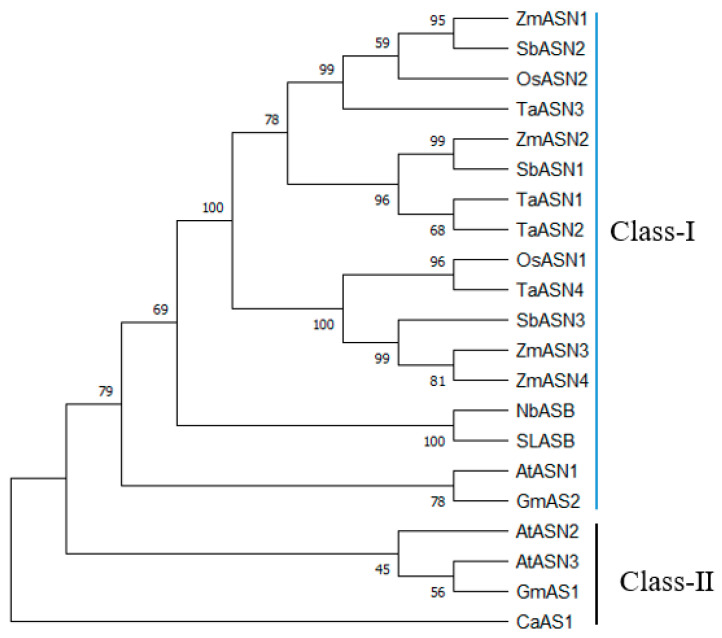
Phylogenetic relationships of As genes from different species. NCBI accession numbers for different species: ZmASN1 (NP_001352587.1), SbASN2 (XP_020942651.1), OsASN2 (NP_001051086.1), TaASN3 (XP_044468791.1), ZmASN2 (NP_001350932.1), SbASN1 (XP_002465143.1), TaASN1 (XP_044479562.1), TaASN2 (XP_044492261.1), OsASN1 (NP_001059983.2), TaASN4 (XP_047302298.1), SbASN3 (XP_002465144.1), ZmASN3 (NP_001352588.1), ZmASN4 (XP_008664167.1), NbASB (XP_019500180.1), SLASB (XP_004247669.1), AtASN1 (NP_186807.1), GmAS2 (XP_003518214.1), AtASN2 (NP_186808.1), AtASN3 (NP_186809.1), and GmAS1 (XP_003518216.1) CaAS1 (XP_016559698.1).

AS in plants is essentially all AS-B. Based on amino acid sequence homology and evolutionary relationships, they can be further divided into two types: Class I and Class II, with each subclass divisible into dicot and monocot subgroups [23]. Class I is found in both dicots (e.g., *Arabidopsis*, soybean) and monocots (e.g., rice, wheat), while Class II is primarily found in monocots (e.g., rice, wheat). To date, *AS-B* genes have been reported and cloned from numerous plants. The encoded protein sequences typically consist of 579–591 amino acids with a molecular weight of approximately 65 kDa, and their functions are being progressively reported. Furthermore, AS-B amino acid sequences exhibit high homology across different plant species (Figure 2), with differences mainly manifesting in the last 30–40 amino acids at the C-terminus [24].

## 4. Expression Characteristics of Asparagine Synthetase Genes

The structural features of AS proteins are closely related to their expression regulation and physiological functions. The N-terminal glutamine amidotransferase domain and the C-terminal synthetase domain contained in the AS-B protein not only determine its catalytic mechanism (relying on glutamine as the amide donor) but may also influence its subcellular localization, stability, and interactions with regulatory proteins. For example, conserved residues within these domains (such as Cys1, His101, and Asp29) are crucial for enzymatic activity and substrate specificity, which may serve as the functional basis for the differential expression of different AS isozymes in specific tissues or under stress conditions. Furthermore, AS proteins classified into Class I and Class II based on sequence homology and evolutionary relationships may possess differentiated cis-acting elements in their promoter regions, allowing them to respond to distinct endogenous signals or environmental stresses, ultimately leading to divergent expression patterns. Therefore, the structural classification of AS not only reflects differences in catalytic properties but also provides a molecular basis for its differential expression during development and under stress conditions. The expression of As genes in plants exhibits variation not only among different species but also across different tissues within the same species, and it can be induced by external environmental cues [25]: Typically, *AS-B* gene expression is high in young, metabolically active tissues with high nitrogen demand, such as root tips, shoot apices, and young leaves [17,26]. Abundant *AS-B* transcripts are found in root meristematic cells and shoot apices of *Arabidopsis*, supporting the high nitrogen demand for rapid cell division and elongation [17]. Rice *OsASN1* is highly expressed in mesophyll cells, promoting carbon–nitrogen balance [27]; mulberry *MaAS* expression peaks in female flowers during full bloom, potentially related to pollen development and ovule formation; wheat *TaASN2* expression increases during late seed development, participating in nitrogen transport, while expression is lower in mature tissues like old leaves [28,29]. Asn, synthesized by AS-B, serves as a crucial nitrogen transport and storage form, providing sufficient nitrogen for cell division, elongation, and construction in young tissues, ensuring rapid growth and development [30]. In leaf tissues, *AS-B* expression is concentrated in mesophyll cells, particularly those surrounding vascular bundles. These cells are involved in both CO_2_ fixation/carbohydrate synthesis during photosynthesis and nitrogen metabolism. *AS-B* expression here helps integrate photosynthetic carbon skeletons with nitrogen to synthesize Asn, achieving carbon–nitrogen balance and facilitating the transport of excess nitrogen to other tissues. In contrast, *AS-B* expression is relatively low in mature tissues like old leaves and stem bases, due to reduced growth rates, lower nitrogen demand, and diminished nitrogen metabolic activity [11].

Changes in the external growth environment significantly influence *AS-B* gene expression levels, primarily involving nitrogen stress, drought and osmotic stress, low-temperature and oxidative stress, heavy metals, nutrient deficiencies, and exogenous hormone signaling. Regarding nitrogen transport, ample external nitrogen sources promote *AS-B* expression in roots and shoots [27]. In *Arabidopsis*, exogenous organic nitrogen sources induce *AtASN1* mRNA levels, while *AtASN2* expression shows no significant difference [21]. Transferring soybean roots from nitrate medium to nitrogen-free medium significantly reduces the expression of As-related genes, whereas returning them to nitrate medium restores expression to previous levels, indicating nitrate induction of *ASN* gene expression in soybean roots [31]. In rice hydroponic experiments, increasing nitrate or ammonium concentrations significantly elevates *ASN-B* transcript levels in roots and leaves, increasing asparagine synthesis, which helps convert excess nitrogen into Asn for storage, preventing waste or toxicity. Conversely, under nitrogen deficiency, plants finely regulate *AS-B* expression for efficient nitrogen use: upregulating *AS-B* in roots prioritizes limited nitrogen for asparagine synthesis and transport to shoots, while partially suppressing *AS-B* expression in shoots reduces unnecessary nitrogen consumption, ensuring supply to actively growing tissues [27].

Beyond nitrogen, other environmental stresses affect *AS-B* expression. Under drought stress, *AS-B* expression alters in many plants. For example, wheat leaves show rapid upregulation of *AS-B* expression during drought, likely because water deficit increases osmotic stress, and increased synthesis of Asn, an important osmolyte, helps maintain cellular osmotic balance and protect against damage. Accumulated Asn also provides a nitrogen source for recovery post-stress [32]. Under low-temperature stress, rapeseed *AS-B* expression is upregulated, enhancing frost resistance, as Asn accumulation promotes synthesis of ice-inhibition substances and antioxidants, reducing intracellular ice formation risk and mitigating damage [33]. Osmotic stress and exogenous ABA also induce wheat *ASN1* transcription, suggesting salt and osmotic stress induction may involve ABA signaling, though the precise mechanism affecting hormone signaling remains unelucidated [34]. Mulberry *MaAS* is upregulated under salt stress, potentially via an ABA-dependent pathway activating the ABRE element in its promoter [35]; *NbASN* expression in *N. benthamiana* significantly increases upon H_2_O_2_ treatment, generating Asn to scavenge ROS and reduce damage [2]; tobacco root *ASN* expression significantly increases under boron deficiency, primarily functioning to maintain cell wall structure [36,37]. Regarding exogenous hormones, ethylene treatment of soybean leaves significantly increases *AS-B* transcript levels and Asn accumulation by more than half; ethylene likely enhances *AS-B* expression by activating EIN3/EIL1 transcription factor activity, which binds the ERE element in the *AS-B* promoter [38,39].

In summary, *AS* gene expression is influenced by external environmental stresses and exhibits internal tissue specificity, underscoring its importance in plant growth and development. The molecular mechanisms underlying stress-induced differential expression of *AS-B* remain unclear. Current reports indicate regulation primarily through promoter cis-acting elements and transcription factor networks: the wheat *TaASN2* promoter contains an NRE motif bound by NLP transcription factors, regulating its transcription [40,41]; the rice *OsAS1* promoter contains DRE and HSE elements responsive to drought and heat, binding DREB and HSF transcription factors, thus activating *ASN* expression under stress [42]; similarly, the mulberry *MaAS* promoter contains ABRE, ERE, and SARE elements responsive to ABA, ethylene, and SA, respectively, influencing *AS-B* transcription [38]; *Arabidopsis* NLP7 directly activates *AtASN1* expression, regulating nitrogen signaling, while mustard MsILR3 binds the G-box element in the *MsMIOX2* promoter, indirectly affecting *AS-B* expression [43,44].

## 5. Biological Functions of Asparagine Synthetase Genes

### 5.1. The Asparagine Synthetase Gene Modulates Plant Growth and Development

As is a key node in the plant nitrogen metabolic network. Its core function is catalyzing the synthesis of Asn from aspartate and glutamine, involved in nitrogen uptake, transport, and recycling [45]. During nitrogen uptake, when soil nitrogen sources (e.g., nitrate, ammonium) are limited, upregulated *AS-B* expression in roots promotes the conversion of scarce absorbed nitrogen into Asn. Due to its high N:C ratio and stability, Asn is efficiently transported via the phloem to actively growing shoot tissues (e.g., young leaves, shoot apices), meeting growth demands [30]; during nitrogen recycling, upon leaf senescence or nutrient deprivation, upregulated *AS-B* in senescing leaves converts amino acids from protein degradation (e.g., Glu, aspartate) into Asn, which is transported to seeds or new organs for reuse, minimizing nitrogen loss [46,47]. Thus, AS primarily influences seed germination and plant growth by affecting nitrogen transport and storage. Supported by theory, numerous studies have been reported. During seed germination, nitrogen stored as Asn is converted to Arg as a storage protein; upon germination, nitrogen from Arg is reactivated to Asn, promoting germination and growth [45]. ASN activity is barely detectable in dry mung bean seeds but increases significantly after imbibition, constituting most of the reduced nitrogen in cotyledons, increasing tens of times compared to controls [48]. In crops, *ASN-B* expression levels correlate positively with nitrogen use efficiency [45]. For example, transgenic rice overexpressing *ASN-B* under low nitrogen shows 15–20%, 10–15%, and 20–25% increases in biomass, grain yield, and nitrogen uptake efficiency, respectively, compared to wild-type, with significantly higher Asn levels. Superior *AS-B* alleles in wheat (e.g., promoter variants with an added NRE element) increase *AS-B* expression by over 30% under low nitrogen, significantly enhancing NUE compared to common alleles [49,50]. Mutation of *OsASN1* in rice significantly reduces its expression and tiller bud number [8]. Additionally, *osasn1* mutants show slight increase in shoot length and slight decrease in root length compared to wild-type [27]. These studies indicate *AS* genes are important candidates for improving NUE and reducing fertilizer use.

As an important form of nitrogen storage and long-distance transport in plants, asparagine is crucial for the growth and development of maize [51]. In rice, OsASN1 is essential for asparagine-dependent development, as its mutants exhibit changes in plant height, root length, and tiller number, along with a sharp decline in asparagine content, although total nitrogen uptake remains unaffected [5]. This suggests that ZmASN1 may play a similarly critical role in early growth, nutrient allocation, and reproductive development (such as grain formation) of maize. In Arabidopsis, the asparagine synthetase encoded by ASN1, expressed in floral organs, contributes to nitrogen filling in seeds [52].

During vegetative growth, plants allocate nitrogen assimilated and accumulated in source organs for protein synthesis during leaf expansion, tissue elongation, and seed development. Asp, Asn, Gln, and Glu are major nitrogen compounds in phloem sap, transported to sink tissues as nitrogen donors for new amino acid synthesis [17]. Arabidopsis lines overexpressing AtASN2 show enhanced growth phenotypes and increased expression in developing leaves and stems, but Asn levels are higher in sink organs (flowers, pods) than source organs (leaves, stems), due to enhanced transport of leaf-synthesized Asn via phloem to sinks. In contrast, asn mutants exhibit growth defects, NH_4_^+^ accumulation, reduced Asn in phloem sap, delayed yellowing, weakened Asn synthesis in sources, and impaired Asn phloem transport [2]. Thus, during vegetative stage, AtASN2 in Arabidopsis leaves participates in nitrogen assimilation and reloads synthesized Asn into the phloem for nitrogen redistribution to metabolic sinks [17]. In potato (*Solanum tuberosum* L.), Asn and Gln are preferentially utilized in developing leaves, playing a major role in nitrogen enrichment and redistribution in the phloem [53,54]. In various plant species, the assimilated nitrogen carriers are initially transported to the shoots via the xylem rather than the phloem: in the model plant Arabidopsis thaliana, nitrate assimilated by the roots is loaded into xylem vessels under the mediation of transporters such as NRT1.5 and directionally transported upward to the above-ground organs, including stems and leaves [55]; in gramineous crops such as wheat and rice, as well as dicotyledonous vegetables such as tomato and cucumber, organic nitrogen compounds (e.g., amino acids and amides) synthesized in the roots are also preferentially allocated from underground to above-ground parts through the xylem in the initial stage [56]; relevant physiological studies have further confirmed that the initial nitrogen transport pathway of field crops such as maize and soybean also conforms to this pattern, where the phloem only participates in nitrogen recycling and bidirectional translocation between sources and sinks in the subsequent stages [57]. Asn is a major free amino acid in potato tubers; isotopic labeling revealed tubers synthesize Asn directly via AS-B, rather than importing it from leaves [58], further confirming AS involvement in nitrogen assimilation. Loss of function of the rice *RST1* gene, which directly suppresses the expression of aspartate synthetase 1 (*OsAS1*), increases *OsAS1* expression, promotes aspartate production, avoids excess ammonium accumulation, and improves nitrogen utilization. *RST1* underwent directional selection during domestication; the superior haplotype *RST1*Hap III reduces its transcriptional repression activity and contributes to salt tolerance and grain weight [8]. These studies demonstrate that AS genes enhance plant growth, development, and yield effects in different species primarily by influencing nitrogen synthesis and transport.

### 5.2. The Asparagine Synthetase Gene and Abiotic Stress in Plants

**Figure 3:**
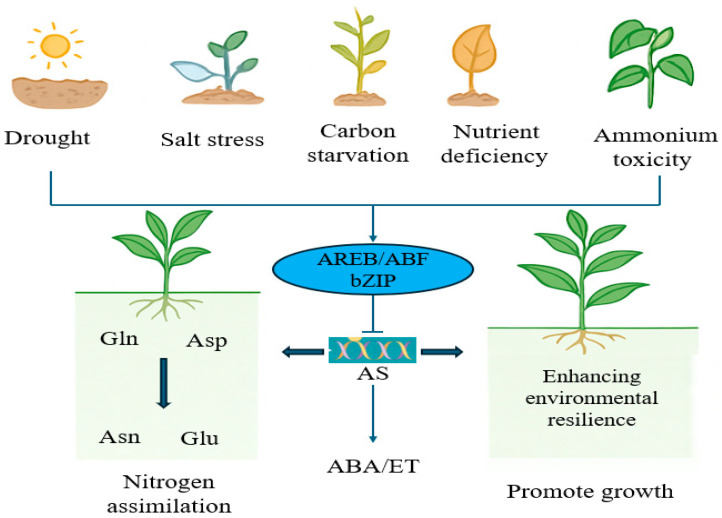
Function of asparagine synthetase gene in plant abiotic stress.

The expression of As genes is induced by various abiotic stresses, including “carbon starvation”, nitrogen stress/ammonium toxicity, nutrient deficiency, temperature stress, and heavy metal stress. Research shows varying response patterns of *AS* genes to stress across plants. In *Arabidopsis*, dark treatment rapidly induces *ASN1* expression and Asn synthesis, and *ASN1* is proven to be key in carbon starvation response. In sunflower, high salt, osmotic, and heavy metal stress induce expression of *HAS1* and *HAS1.1*, but not *HAS2* [59]. Similarly, rice *OsAS1* expression is induced by salt and osmotic stress, while *OsAS2* shows no significant difference. Further research revealed that upregulation of *OsAS1* under salt stress is primarily regulated by the transcription factor RST1, which binds the *OsAS1* promoter and represses its expression; salt stress alleviates this repression, leading to *OsAS1* upregulation [8,60]. These results indicate that under drought and salt stress, not only does osmotic stress reduce carbon fixation, but the stress itself directly or indirectly increases *ASN* expression, primarily for nitrogen redistribution to maintain cellular osmotic pressure balance and nitrogen homeostasis [61,62]. When ammonium is the sole N source or nitrification is inhibited, ammonium accumulates in plants, leading to toxicity. ASN, utilizing Gln or NH_4_^+^ as donors to synthesize Asn, serves as a key enzyme for ammonium assimilation and detoxification. In rice and *Arabidopsis*, ammonium accumulation significantly increases *ASN* gene expression, generating Asn to reduce free ammonium concentration and alleviate cytotoxicity [63,64]. In tobacco, *AS-B* expression is significantly upregulated under boron deficiency, mainly concentrated in roots, with no significant change in leaves, suggesting soluble boron deficiency may specifically regulate root *AS-B* expression. Additionally, *AS-B* expression changes significantly under drought and low temperature in tobacco [65]. These findings indicate AS participates in plant responses to different stresses with diverse biological functions. The specific regulatory mechanisms for tissue-specific *ASN* expression differences under various stresses primarily involve: (1) Maintaining nitrogen remobilization and utilization: Under stress, protein synthesis decreases while degradation increases, releasing free amino acids. Asn reincorporates nitrogen from these into Asn, allowing nitrogen transfer from senescing or stressed tissues to actively growing tissues, providing nutrients for repair and reproduction while reducing waste [66]. (2) Involvement in osmotic adjustment and ROS balance: Glu serves as the primary precursor for the biosynthesis of glutathione (GSH), a major cellular antioxidant. The revised text delineates this process: stress-induced AS activity elevates Glu production, which in turn fuels the glutathione biosynthesis pathway (Glu-Cys-Gly) [67]. The enhanced GSH pool bolsters the cellular capacity for ROS scavenging via the ascorbate–glutathione cycle, thereby mitigating oxidative damage [68]. Concurrently, stress-induced upregulation of AS supplies increased substrate (Glu) for the proline-synthesizing enzymes P5CS and P5CR, leading to proline accumulation. Proline functions as a potent and well characterized osmoprotectant that stabilizes proteins and cellular structures under osmotic stress [69,70]. Asn-mediated nitrogen metabolism reprograms the supply of precursors for other osmolytes like proline, indirectly affecting NO and ROS metabolism, mitigating oxidative damage [71]. (3) Integrating stress metabolic pathways: *ASN* expression is regulated by multiple transcription factors core to key stress-signaling pathways. Under carbon starvation, *ASN1* expression is regulated by bZIP transcription factors; under drought, the *ASN* promoter region can be recognized by AREB/ABF factors [72], leading to transcriptional activation or repression, further influencing AS expression (Figure 3).

In summary, AS expression under diverse abiotic stresses converges on a few core regulatory nodes that link environmental perception to metabolic adaptation. Firstly, nitrogen signaling is a primary driver, where transcription factors like NLP directly activate *ASN* genes in response to nitrogen status, facilitating ammonium detoxification and remobilization. Secondly, hormone- and stress-responsive transcription factors serve as key integrators: ABF/AREB proteins mediate ABA-dependent regulation during drought and osmotic stress; DREB proteins and HSFs link AS expression to dehydration and temperature extremes, respectively; and RST1-like repressors modulate salt-responsive expression. Thirdly, redox signaling, often involving H_2_O_2_, induces *ASN* expression, aligning nitrogen metabolism with the need for antioxidant synthesis. Ultimately, these regulatory inputs funnel through the AS enzyme to execute three pivotal downstream functions: (1) nitrogen recycling and transport, (2) osmotic adjustment via Asn and its derivative proline, and (3) reinforcement of antioxidant and defense hormone SA pathways via Glu provision. Thus, AS acts as a central metabolic effector positioned at the intersection of multiple stress-signaling networks, enabling coordinated resource reallocation toward stress tolerance.

### 5.3. Asparagine Synthetase Gene in Bacterial Infection

Similarly, a Zhejiang University team found that 2,4-di-tert-butylphenol (2,4-DTBP) secreted by the symbiotic fungus *Aspergillus cvjetkovicii* activates the AS-B-mediated nitrogen metabolism pathway, enhancing rice resistance to *Rhizoctonia solani*. This phyllosphere symbiont–plant pathogen interaction, mediated by chemical signals regulating AS-B, provides new insights into AS-B’s ecological role and potential for developing microbial agents [73]. Expressing the *Physcomitrium patens ASN* gene in *Arabidopsis* increases Asn content and reduces bacterial (*Pseudomonas syringae*) growth compared to controls [30]. Subsequent *asn1/asn2* double mutants show increased susceptibility and enhanced bacterial multiplication, indicating AS genes positively regulate resistance against *P. syringae* in *Arabidopsis* [74]. In tomato, silencing *SLASN* reduces resistance to *P. syringae*; mechanistic studies revealed lower SA levels and downregulated SA pathway genes in mutants, explaining how AS-B enhances resistance via SA signaling [75]. Beyond hormones, silencing *OsAS1* in rice reduces ROS burst upon pathogen inoculation, indicating AS-B is essential for early defense responses [76]. These studies suggest AS-B does not directly inhibit pathogen growth but acts as an intermediary affecting downstream defense signaling or integrates nutrient signals to coordinate resource allocation towards defense, indirectly impacting pathogen growth.

### 5.4. The Asparagine Synthetase Gene Confers Virus Resistance in Plants

**Figure 4:**
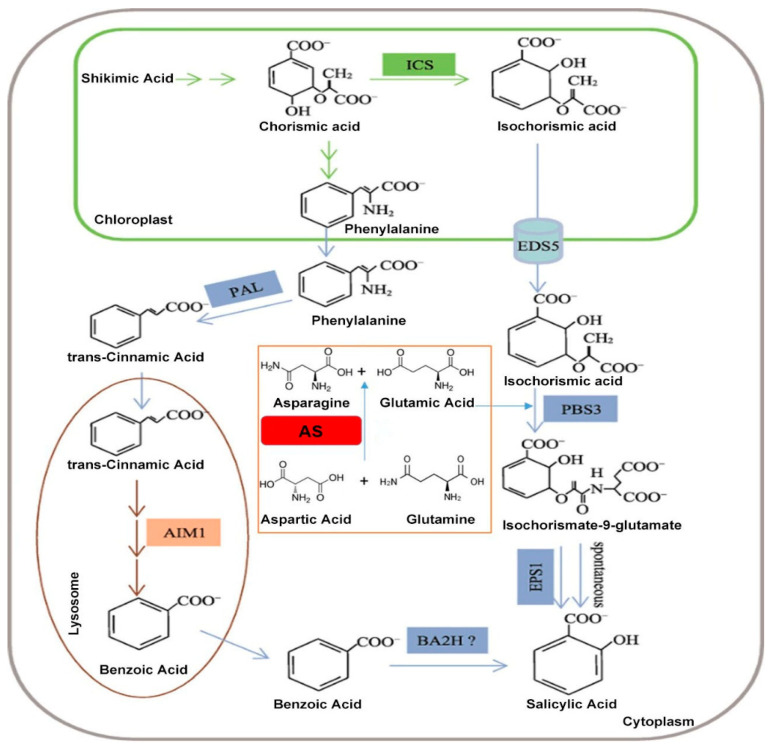
Simplified scheme of possible pathways for the biosynthesis of SA in *Arabidopsis*: In *Arabidopsis*, SA is primarily synthesized via the isochorismate pathway. This review adds a key focus on the origin of Glu in plants, which can be produced through AS synthesis. Subsequently, Glu conjugates with isochorismate and, under the action of PBS3, generates isochorismate-9-Glu. The relevant enzymes and their abbreviations are as follows: Isochorismate Synthase (ICS), Phenylalanine Ammonia Lyase (PAL), Enhanced Disease Susceptibility 5 (EDS5), avrPphB Susceptible 3 (PBS3), Enhanced Pseudomonas Susceptibility 1 (EPS1), Abnormal Inflorescence Meristem 1 (AIM1), and Benzoic Acid 2-Hydroxylase (BA2H). Adapted from [81].

AS primarily catalyzes the synthesis of Asn and Glu from Asp and Gln in plants. Previously, research focused on its roles in growth, nitrogen metabolism, abiotic stress, and antibacterial defense, with few reports on antiviral activity. However, advancing techniques and pathway elucidation have revealed new functions. Evidence suggests Asn and Glu participate in antiviral defense. In *N. benthamiana*, *NbAS-B* accumulation enhances resistance to Tobacco Mosaic Virus (TMV); mechanistic analysis suggests substrates Asn and Glu can modulate the SA pathway, TMV infection induces SA pathway genes like PR1 and PR2, enhancing resistance. Our lab found that the other catalytic product, Glu, is crucial for SA pathway function in this context. Inhibiting Glu with DFMTI abolished the antiviral effect of AS-B, indicating dependence on Glu-induced SA signaling [19]. The relationship between Glu and SA involves two SA biosynthesis pathways: the isochorismate synthase (ICS) and phenylalanine ammonia-lyase (PAL) pathways [77,78], both starting from chorismate in chloroplasts. (1) PAL pathway: Isotope labeling in tobacco showed Phe conversion via trans-cinnamic acid (t-CA) and benzoic acid to SA, catalyzed by PAL and AIM1, with benzoic acid potentially being hydroxylated by an unidentified BA2H. (2) ICS pathway: Chorismate is converted to isochorismate by ICS1, then requires EDS5 (transport to cytoplasm) and PBS3 (an aminotransferase). PBS3, with key residues for Glu-binding (Lys428, Lys146), Mg^2+^-binding (Glu329), and ATP/AMP interaction (Ser328, Asp398, Lys550), catalyzes the formation of an isochorismate-9-Glu adduct (ISC-9-Glu), which spontaneously decomposes or is catalyzed by EPS1 to produce SA [79,80,81,82,83] (Figure 4). While PAL is prominent in rice/tobacco and ICS in Arabidopsis, both may operate to varying degrees in different plants. Suppressing ICS homologs in tomato and N. benthamiana reduces SA accumulation [84,85,86], but ICS pathway evidence outside Brassicaceae needs strengthening.

Additionally, mechanical damage or herbivory releases Glu from damaged cells, creating a gradient that activates Glu receptor-like (GLR) channels, leading to Ca^2+^ influx and membrane potential changes, triggering downstream responses [87]. In *N. benthamiana*, Glu binding to GLR3.3 induces Ca^2+^ influx, promoting SA signaling and immunity. Thus, Glu involvement in plant immunity primarily occurs through these two pathways, ultimately inducing SA signaling. Therefore, exploring the Glu-SA link in *N. benthamiana* is crucial for understanding Glu’s role in disease resistance.

AS balances plant growth and stress tolerance by functioning as a central metabolic node in nitrogen homeostasis, controlling the allocation and remobilization of nitrogen via Asn. Under favorable conditions, enhanced AS activity channels assimilated nitrogen into Asn, which is transported to sink tissues—such as meristems and developing seeds—to support protein synthesis and biomass accumulation, thereby promoting growth. Under stress, hormones such as ABA and SA reprogram AS expression, shifting its role toward stress adaptation. In this state, AS mediates: nitrogen remobilization and protection by retrieving nitrogen from degraded proteins and converting it to Asn for redistribution to defense or repair sites; and osmotic adjustment and defense synthesis, with Asn serving as a compatible osmolyte and its product Glu acting as a precursor for glutathione, proline, and SA, a key defense hormone. Thus, AS does not equally promote growth and stress resistance simultaneously, but rather acts as an environmentally responsive metabolic switch. Under optimal conditions, it directs nitrogen toward growth; under stress, it reallocates resources to defense and survival pathways. Notably, Glu production directly links AS activity to SA biosynthesis, establishing a molecular interface that integrates nitrogen metabolism with systemic disease resistance.

In addition to transcriptional and metabolic regulation, the activity, stability, and subcellular localization of AS may be precisely modulated by post-translational modifications, offering a rapid and reversible regulatory mechanism for cellular signal responses. Although direct evidence for PTMs of plant AS proteins remains limited, post-translational modifications are nevertheless an important factor influencing protein function. Phosphorylation represents a key potential mechanism for regulating AS function: kinases activated by energy-sensing pathways (e.g., SnRK1 under carbon starvation) or stress-activated MAPKs may phosphorylate AS, thereby affecting its catalytic efficiency, interaction with binding partners, or intracellular trafficking. Ubiquitination may be involved in AS protein turnover, mediated by E3 ubiquitin ligases responsive to hormonal signals (e.g., ABA, ethylene) or nitrogen status, leading to targeted degradation of AS via the 26S proteasome pathway and enabling rapid clearance when enzymatic activity is no longer required. Furthermore, redox-related modifications such as S-nitrosylation or cysteine oxidation under oxidative stress may transiently modulate AS activity, directly linking its function to cellular redox homeostasis. Integrating these potential PTM mechanisms with the aforementioned transcriptional regulatory networks could enable plants to establish a multi-layered, dynamic regulatory system for asparagine synthesis, thereby enhancing their agility in adapting to environmental changes.

## 6. Conclusions

AS, particularly the plant-specific AS-B isoform, functions as a pivotal metabolic integrator, orchestrating the fundamental trade-off between nitrogen utilization for growth and investment in stress resilience. Moving beyond its classic role in nitrogen transport and storage, AS-B emerges as a key regulatory node. Its enzymatic products—Asn for nitrogen distribution and Glu as a signaling precursor—directly couple nitrogen metabolism with defense activation, notably through supporting SA biosynthesis for pathogen resistance. Furthermore, AS-B expression is modulated by diverse abiotic stresses and hormones, enabling metabolic reprogramming that enhances stress adaptation. This positions AS-B as a central processor of internal nutrient status and external cues, dynamically allocating resources to optimize fitness. Future perspectives center on deciphering the precise molecular mechanisms controlling AS-B activity and spatial regulation. Harnessing this knowledge to engineer “smart” AS-B alleles that optimize the growth–defense balance under specific environments holds significant promise for developing resilient crops with improved nitrogen use efficiency and sustainable yield stability.

## Data Availability

The original contributions presented in this study are included in the article. Further inquiries can be directed to the corresponding authors.

## Abbreviations

2,4-DTBP	2,4-di-tert-butylphenol
ABA	Abscisic acid
ABRE	ABA-Responsive Element
AIM1	Abnormal Inflorescence Meristem 1
AREB/ABF	ABRE-Binding Protein/Factor
AS	Asparagine Synthetase
Asn	Asparagine
Asp	Aspartate
BA2H	Benzoic Acid 2-Hydroxylase
DFMTI	Difluoromethylthioimidazole-2-thione
DRE	Dehydration-Responsive Element
DREB	Dehydration-Responsive Element Binding protein
EC	Enzyme Commission number
EDS5	Enhanced Disease Susceptibility 5
EPS1	Enhanced Pseudomonas Susceptibility 1
GLR	Glu Receptor-Like
Gln	Glutamine
Glu	Glutamate
HSE	Heat Shock Element
HSF	Heat Shock Factor
ICS	Isochorismate Synthase
NLP	NIN-Like Protein
NRE	Nitrogen-Responsive Element
NUE	Nitrogen Use Efficiency
PAL	Phenylalanine Ammonia-Lyase
PBS3	avrPphB Susceptible 3
PCD	Programmed Cell Death
PR	Pathogenesis-Related
ROS	Reactive Oxygen Species
SA	Salicylic Acid
